# Mineralocorticoid Receptor Antagonists Eplerenone and Spironolactone Modify Adrenal Cortex Morphology and Physiology

**DOI:** 10.3390/biomedicines9040441

**Published:** 2021-04-20

**Authors:** Sofia S. Pereira, Liliana Carvalho, Madalena M. Costa, Armindo Melo, Isabel M. P. L. V. O. Ferreira, Celso E. Gomez-Sanchez, Mariana P. Monteiro, Gavin Vinson, Duarte Pignatelli

**Affiliations:** 1 Department of Anatomy and Unit for Multidisciplinary Biomedical Research (UMIB) of Instituto de Ciências Biomédicas Abel Salazar (ICBAS), University of Porto, 4050-313 Porto, Portugal; sspereira@icbas.up.pt (S.S.P.); mcosta@icbas.up.pt (M.M.C.); mpmonteiro@icbas.up.pt (M.P.M.); 2 Department of Experimental Biology, Faculty of Medicine, University of Porto, 4200-319 Porto, Portugal; lsscarvalho.fmup@gmail.com; 3 Department of Environmental Health, National Institute of Health Dr. Ricardo Jorge, 4000-055 Porto, Portugal; armindo.melo@insa.min-saude.pt; 4 Laboratório Associado Para a Química Verde (LAQV/REQUIMTE), University of Porto, 4050-313 Porto, Portugal; isabel.ferreira@ff.up.pt; 5 Laboratory of Bromatology and Hidrology, Department of Chemical Sciences, Faculty of Pharmacy, University of Porto, 4050-313 Porto, Portugal; 6 Endocrinology Section, G.V. (Sonny) Montgomery VA Medical Center, Montgomery, AL 36109, USA; cgomez-sanchez@umc.edu; 7 Department of Medicine, University of Mississippi Medical Center, Jackson, MS 39216, USA; 8 The School of Biological and Chemical Sciences, Queen Mary University of London, London E1 4NS, UK; g.p.vinson@qmul.ac.uk; 9 Cancer Signalling & Metabolism Group, Instituto de Investigação e Inovação em Saúde, University of Porto, 4200-135 Porto, Portugal; 10 Cancer Signalling & Metabolism Group, Institute of Molecular Pathology and Immunology of the University of Porto (IPATIMUP), 4200-135 Porto, Portugal; 11 Department of Endocrinology, Hospital S. João, 4200-319 Porto, Portugal

**Keywords:** adrenal cortex, mineralocorticoid receptor antagonists, eplerenone, spironolactone, steroids

## Abstract

Mineralocorticoid receptor antagonists (MRAs) are a class of anti-hypertensive drugs that act by blocking aldosterone action. The aim of this study was to evaluate whether the MRAs spironolactone and eplerenone influence adrenal cortical physiology and morphology. Spontaneous hypertensive rats (SHR, *n* = 18) and normotensive rats (WKY, *n* = 18) were randomly exposed to a daily dose of spironolactone (*n* = 6), eplerenone (*n* = 6), or no drug (*n* = 6) over 28 days. After that, aldosterone, corticosterone, and 11-deoxycorticosterone plasma concentrations were quantified. Adrenal glands were subjected to morphological analysis to assess lipid droplets content, capsular width, cell proliferation, and steroidogenic proteins expression. The adrenal cortex in untreated SHR showed higher lipid droplet content as than in WKY. In SHR, MRA treatment was associated with higher circulating aldosterone levels and Ki-67 expression in aldosterone-secreting cells. In WKY, the only difference observed after MRA spironolactone treatment was a narrower capsule. There was no difference in abundance of steroidogenic enzyme between groups. In conclusion, MRAs modify adrenal gland function and morphology in SHR. The effects observed within the adrenal glomerulosa with aldosterone-secreting cell proliferation and higher circulating aldosterone levels suggests that MRA treatment provokes activation of the renin angiotensin system. The prognostic value of hyperaldosteronism secondary to MRAs blockade requires further investigation.

## 1. Introduction

The mineralocorticoid aldosterone is produced in the zona glomerulosa of the adrenal cortex. Its synthesis and secretion are primarily regulated by the renin-angiotensin system (RAS) and its primary function is to regulate sodium reabsorption in the distal convoluted tubule of the kidney [1]. Aldosterone effects are mediated through the mineralocorticoid receptor and its blockade with mineralocorticoid receptor antagonists (MRAs) is used for the treatment of hypertension and heart failure. There are two MRAs, spironolactone and eplerenone, approved by the Food and Drug Administration (FDA) [2,3,4]. MRAs inhibit aldosterone from binding to its receptor and make it transcriptionally inactive. Consequently, aldosterone-induced sodium reabsorption is prevented, thus leading to blood pressure decrease [5,6].

Spironolactone was the first MRA to be commercially available for the treatment of hypertension and heart failure. Spironolactone is considered a non-selective MRA since it also binds to progesterone or androgen receptors with potentially adverse effects. In contrast, eplerenone is a more selective MRA, with a lower affinity for androgen and progestin receptors, thus reducing the possibility of side effects [2,7].

Additionally, prolonged treatment with spironolactone has been shown to alter adrenal cortex morphology, leading to the appearance of round, laminated cytoplasmic inclusions surrounded by a clear halo, the so called “spironolactone bodies” [8,9,10]. However, the functional significance of “spironolactone bodies” remains to be elucidated.

In contrast, few studies have explored the possible effects of eplerenone on adrenal cortex morphology although a previous study was unable to document the appearance of any morphological features similar to “spironolactone bodies” in the human adrenal cortex after eplerenone exposure [9].

Therefore, our aim was to explore the effects of MRAs, spironolactone and eplerenone, on adrenal cortex physiology and morphology.

## 2. Materials and Methods

### 2.1. Animals and Experimental Design

Adult male normotensive Wistar Kyoto (WKY) rats (*n* = 18) and male Spontaneously Hypertensive (SHR) rats (*n* = 18) were maintained under controlled temperature (21–23 °C), 60% relative humidity and constant photoperiod conditions (12 h light, 12 h dark) with ad libitum access to food and water. Housing and experimental treatment of the animals were in accordance with the Guide for the Care and Use of Laboratory Animals from the Institute for Laboratory Research. All procedures were approved by the institutional Ethics Board for Animal Research (ORBEA_3_2013/0110) and followed the European Union laws on animal protection (86/609/EC) and the Portuguese legislation for the same purpose (DL 113/2013).

After 1 week of acclimatization, WKY and SHR rats were randomized into three groups (*n* = 12) comprising WHY (*n* = 6) and SHY (*n* = 6). The first group was treated with eplerenone (50 mg/kg/day, Inspra, Pfizer, New York, NY, USA) via oral administration, the second group was treated with spironolactone (100 mg/kg/day, Aldactone, Pfizer, New York, NY, USA) and the third group was untreated. as control. Rats received treatment for a 28-day period, during which systolic and diastolic blood pressures were monitored by using the tail-cuff method every seven days.

At the end of this period, rats were deeply anesthetized using Isoflurane (Abbott Laboratories Limited, Maidenhead, UK) and then sacrificed by cardiac puncture. Blood samples were collected into EDTA tubes (BD Vacutainer, Thermo Fisher Scientific, Waltham, MA, USA). Adrenal glands were dissected and removed for histological analysis. Right adrenal glands were fixed in 4% buffered formaldehyde (Panreac Applichem, Darmstadt, Germany) for 24 h and then submitted to automatic tissue processing procedures for light microscopy. Left adrenal glands were embedded in optimum cutting temperature (OCT) (Cryomatrix embedding resin; Thermo Fisher Scientific, Waltham, MA, USA) in a cryomold and rapidly frozen in 2-methylbutane (Merck, Darmstadt, Germany) cooled by dry ice and stored at −80 °C, until use.

### 2.2. Luxol Fast Blue Staining for Spironolactone Bodies Detection

Paraffin-embedded adrenal slices were deparaffinized and rehydrated. Slices were initially stained with a Luxol fast blue solution (ref. S3382, Sigma-Aldrich, Missouri, USA), at 57 °C, for 2 h. Then, tissue slices were differentiated with a solution of 0.05% lithium carbonate (ref. 62470, Sigma-Aldrich, St. Louis, MO, USA) followed by alcohol at 70%. A 0.1% Cresyl violet solution (ref. C0182, Diapath, Martinengo, Italy) was used as a counterstain.

### 2.3. Oil Red O for Lipid Droplet Evaluation

For the detection of adrenal lipid droplets, the Oil red O (ORO) staining was performed in three 12-μm-thick sections of each frozen adrenal glands mounted on adhesive microscope slides Superfrost (Thermo Fisher Scientific, Waltham, MA, USA). The ORO protocol used was similar to the previous described by Mehlem et al. [11].

A solution of ORO 62.5% (ref. O0625, Sigma-Aldrich, St. Louis, MO, USA) in isopropyl alcohol (Merck, Darmstadt, Germany) was made and then diluted 1:1.7 in distillated water. The sections were incubated with this solution for 10 min and then rinsed under running tap water for 6 min. The slides were counterstained with Mayer′s hematoxylin (Merck, Darmstadt, Germany) and mounted with a water-soluble mounting medium (Aquatex, Merck, Darmstadt, Germany).

A minimum of eight fields of each adrenocortical zone were caputured at 200× at least 24 h after the staining, to avoid precipitation of the ORO dye, using a Leica camera (DFC290, Leica, Wetzlar, Germany) coupled to an optical microscope (Zeiss, Oberkochen, Germany). Although the glomerulosa and fasciculata are easily distinguishable to allow morphological analysis, it is difficult to perform an accurate separation of the reticularis from the fasciculata layer. Therefore, the fasciculata analysis was performed in areas remote from the reticularis: the reticularis was not analyzed.

Morphological analysis was performed based in the protocol described by Mehlem et al., using an image processing software FIJI-ImageJ (originated at the National Institutes of Health, USA) with a color deconvolution plugin (vector matrix for the ORO color: R: 0.062340338; G: 0.6408937; B: 0.7650941), that enables separate quantification of differently stained regions—in this case, the area occupied by lipid droplets as a proportion of the whole tissue analyzed [11].

### 2.4. Sirius Red for Capsule Evaluation

Sirius red staining was used to visualize collagen present in the adrenal capsule [12,13]. Briefly, after deparaffinization and rehydration, sections were incubated with Mayer′s Hematoxylin (Merck, Darmstadt, Germany) and Celestine Blue (Merck, Darmstadt, Germany) for 8 min followed by the incubation with 0.1% (*w/v*) Sirius Red (Merck, Darmstadt, Germany) for 1 h, at room temperature. Sections were washed with acidified water, dehydrated, and mounted in a resinous medium.

Slides were scanned using the image acquisition software Olympus VS110 virtual slide scanning system (Olympus, Tokyo, Japan). Images were analyzed using the FIJI as performed for the ORO staining and then the area stained with Sirius red was normalized to the perimeter of each adrenal gland, obtained by using the same software.

### 2.5. Immunohistochemistry Protocol

Immunohistochemistry (IHC) was performed for detection of aldosterone synthetase (CYP11B2), 11β-hydroxylase (CYP11B1), inner zone antigen (IZA, also known as Progesterone receptor membrane component 1 (PGRMC)), and Ki-67.

The CYP11B1 and CYP11B2 antibodies used in this study were provided by Dr. Celso E. Gomez-Sanchez, Medical Center, University of Mississippi, USA, and the IZA antibody was developed by Gavin Vinson’s group at Queen Mary University of London, UK.

IHQ was performed on formalin-fixed paraffin embedded adrenal glands sections, mounted on adhesive microscope slides. Sections were successively deparaffinized, rehydrated in graded alcohols, followed by antigen retrieval. For IZA and Ki-67, slides were incubated with 0.01 M-citrate buffer at pH 6.0 with 0.05% Tween 20 (Sigma-Aldrich, St. Louis, MO, USA), in a microwave oven at 900 W, during 25 min after boiling, and the endogenous peroxidase was blocked with 3% hydrogen peroxide (Merck, Darmstadt, Germany) in methanol (Sigma-Aldrich, St. Louis, MO, USA). For CYP11B1 and CYP11B2, slides underwent 45 min heating in a temperature-controlled water bath (99.9 °C) with ethylenediaminetetraacetic acid (EDTA) (E5134, Merck, Darmstadt, Germany) solution 1 mm at pH 9 with Sodium dodecyl sulfate (SDS, Sigma-Aldrich, St. Louis, MO, USA) 0.05%, and endogenous peroxidase inhibition was performed using hydrochloric acid (Panreac Applichem, Darmstadt, Germany), at 0.02N, for 20 min.

The sections were then incubated overnight at 4 °C with the primary antibodies: Ki-67 (1:500; 27R-14; Cell Marque, Rocklin, CA, USA), IZA (1:100), CYP11B1 (1:500) or CYP11B2 (1:100). For IZA detection, the slices were incubated with a secondary antibody at 1:200 (Polyclonal swine anti-rabbit, Dako, Næstved, Denmark), followed by avidin-biotin peroxidase complexes (1:100, Vector Laboratories, INC., CA, USA), for 30 min.

For Ki-67, CYP11B1, and CYP11B2, immune reaction detection was performed by incubation for 60 min with the commercial Dako REAL™ EnVision™ Detection System (ref. K5007, Dako, Næstved, Denmark). For all proteins, diaminobenzidine (Dako, Næstved, Denmark) was used as the chromogen and hematoxylin as the nuclear counterstain.

Stained slides were scanned as described above and images were analyzed using a morphometric computerized software, the FIJI software. Briefly, a color deconvolution plugin (HDab) was used to separate the stained brown area from the initial image, as previously described [14]. Then, the ratio between the stained area and total area occupied by the adrenal cortex was calculated (% of stained area).

### 2.6. Corticosterone and 11-Deoxycorticosterone Extraction and Quantification

For steroid extraction, each aliquot (200 μL) was transferred into a conic microtube and 20 μL of hydrocortisone (1000 μg/L, Sigma, St. Louis, MO, USA) was added as internal standard (IS). Then, 100 μL of extracting solvent (ACN with 1% formic acid (*v/v*)) (Merck, Darmstadt, Germany) was added along with MgSO4 anhydrous salt (75 mg, Merck, Darmstadt, Germany) and NaCl (20 mg, Merck, Darmstadt, Germany) immediately vortexing the tube for 10 s. Tubes were centrifuged (13,000 rpm for 5 min), the organic upper phase was transferred to a 2 mL vial with a glass insert, and analyzed by LC-MS/MS through injection of 20 μL in each sample.

The separation of corticosterone and 11-deoxycorticosterone was realized with a LC Waters 2695 XE separation module (Waters, Massachusetts, USA). The column used was a core–shell Kinetex C18 column (2.6 μm; 100 × 2.1 mm) kept at 30 °C. Chromatographic separation was performed using a mixture of two eluents: a mobile phase A consisted of 10 mm ammonium acetate/acetic acid (Merck, Darmstadt, Germany), pH 4 and a mobile phase B consisted in methanol. The adopted gradient elution consisted of the following steps: (i) 0–2 min, 20% of B in A; (ii) from 2 to 4 min, 20–90% of B in A increase; (iii) 4–8 min, 90% of B in A; (iv) from 8 to 9 min 90–98% of B in A increase; (v) 98% of B in A kept until 13 min. Afterwards, the column was rinsed and reequilibrated (10 column volumes) for 7 min before the next injection. The mobile phase flow rate was 0.2 mL/min and the injection volume was 20 μL. A Waters Micromass^®^ Quattro micro™ API triple quadrupole mass analyzer (Waters, Massachusetts, USA), equipped with an electrospray ionization source in the negative ion mode was used under the following conditions: (i) 3.00 kV capillary voltage; (ii) 180 °C source temperature, and 350 °C desolvation temperature; (iii) 600 L/h desolvation gas flow; and (iv) 650 V multiplier. The cone and collision gases were nitrogen (≥99.999%) and argon (≥99.999%), respectively. The MRM transition, cone voltage and collision energy for each analyte is shown in Supplementary Table S1 and were initially determined by flow injection analysis, and calibrated with the IS. The dwell time was set at 200 ms. Data acquisition was performed by the MassLynx V4.1 software (Waters, MA, USA).

Linearity was evaluated by calibration curves with six calibration points in the range of 25–250 μg/L for corticosterone and 2.5–25 μg/L for 11-deoxycorticosterone. Precision was determined by repeatability (intraday precision) and intermediate precision (interday precision) of a spiked sample, at 100 μg/L for corticosterone and 10 μg/L for 11-deoxycorticosterone, using three replicates, on each precision day. The recovery tests were also done in the same concentrations.

The limits of detection (LODs) and limits of quantification (LOQs) were calculated based on the calibration curve parameters according to Miller J et al. [15]. Where the LOD was equal to the calculated intercept of the linear regression (a) plus three times the Sy/x and ten times this value, for LOQ.

Good linear response was verified for the steroids analysed (R2 > 0.9997). The presented LODs and LOQs were 0.31 μg/L and 1.04 μg/L, respectively, for corticosterone. For 11-deoxycorticosterone presented LODs and LOQs were 0.25 μg/L and 0.64 μg/L. The obtained percentages of recovery were in the expected range of 80–120% and the RSD values for interday and intraday precision were lower than 13.5%. All validation method parameters are presented in supplementary material in Supplementary Table S2.

### 2.7. Aldosterone Extraction and Quantification

Aldosterone plasma levels were quantified by ELISA using a commercially available Aldosterone kit (ab136933, Abcam, Cambridge, UK), following the manufacturer’s instructions. To increase accuracy, the assay was preceded by an extraction step performed similarly to that for corticosterone and 11-deoxycorticosterone, with the sole difference that extracts were reconstituted in 250 µL of the assay buffer of the aldosterone ELISA kit.

### 2.8. Statistical Analysis

Continuous variables are expressed as mean ± standard error of the mean unless stated otherwise. For continuous variables with a normal distribution, an unpaired *t*-test was used to compare the means of the two groups. For variables that did not pass the normality test, a Mann–Whitney test was used. To compare three or more independent groups, one-way ANOVA followed by a Tukey’s multiple comparisons test were performed for variables with a normal distribution. For variables that did not pass the normality test, three group comparisons were performed using the Kruskal–Wallis test followed by the Dunn’s multiple comparison test. Comparisons of the blood pressure values between the timepoints was performed using a two-way analysis of variance (ANOVA) with Sidak’s post hoc test. Statistical analysis was carried out using the GraphPad software (GraphPad Software, La Jolla, CA, USA). A *p* < 0.05 was considered statistically significant.

## 3. Results

### 3.1. Arterial Blood Pressure

**Figure 1:**
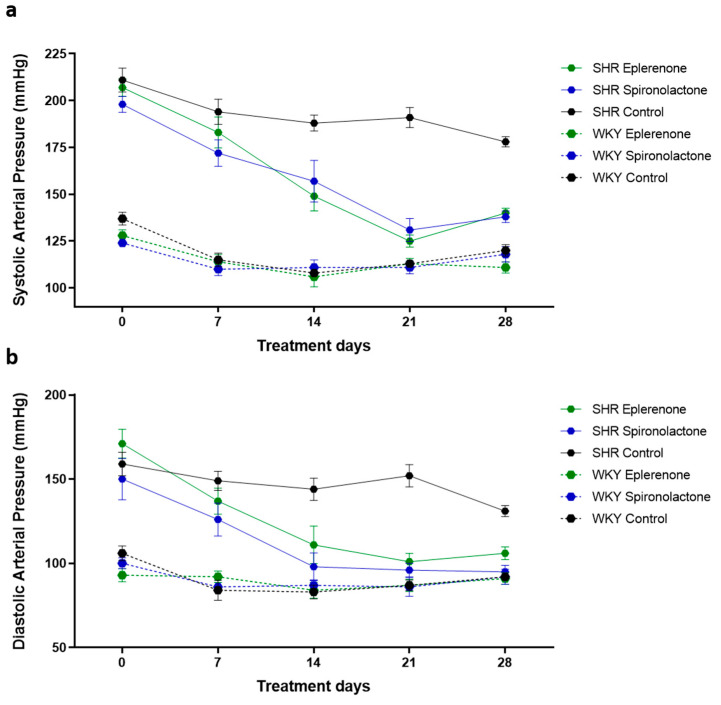
Systolic (**a**) and diastolic (**b**) blood pressure values of Wistar Kyoto (WKY) and Spontaneous Hypertensive rats (SHR), before and 7, 14, 21, and 28 days after treatment with eplerenone or spironolactone. After 21 days of treatment, SHR treated with spironolactone or eplerenone reached blood pressure values similar to those observed in normotensive rats. The arterial blood pressure of the normotensive rats was not affected by MRAs treatment.

Systolic and diastolic blood pressures were measured every seven days from the start of treatment (Figure 1). SHR treated with spironolactone and eplerenone showed a significant decrease in systolic and diastolic blood pressures after the first seven days of treatment when compared with untreated SHR (SHR eplerenone vs. SHR control: *p* < 0.01; SHR spironolactone vs. SHR control: *p* < 0.001). After 21 days of treatment, SHR treated with spironolactone or eplerenone reached blood pressure values similar to those observed in normotensive rats.

The arterial blood pressure of the normotensive rats was not affected by MRAs treatment (*p* > 0.05) (Figure 1).

### 3.2. Adrenal Cortex Morphology

Adrenal cortex morphology was similar between the groups. In particular, no spironolactone bodies were identified in the cortices of treated animals (Supplementary Figure S1).

### 3.3. Lipid Droplets Evaluation

**Figure 2:**
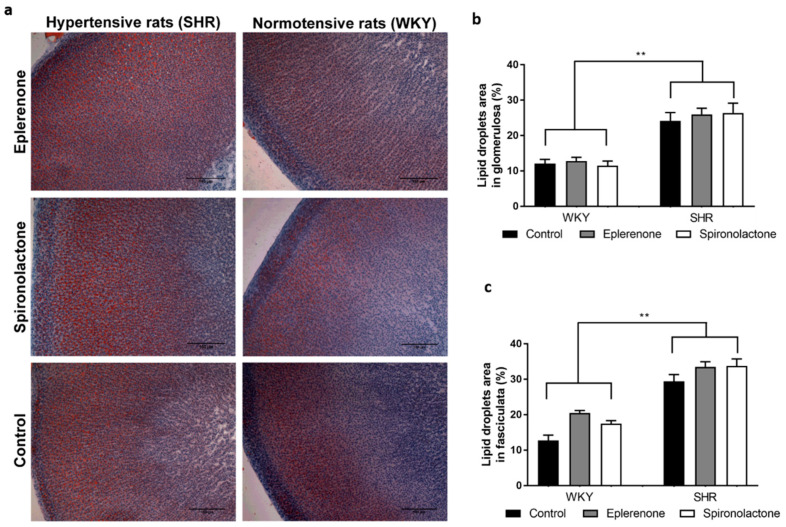
Adrenal glands stained with oil red O (100×) (**a**) and the area of the glomerulosa (**b**) and fasciculata (**c**) layers occupied by lipid droplets. ** *p* < 0.01.

Untreated SHR showed a significantly larger area of the glomerulosa and fasciculata contained lipid droplets compared with WKY rats (*p* < 0.01) (Figure 2). None of the MRAs affected the lipid droplet profile in SHR or WHY rats.

#### 3.3.1. Adrenal Capsule Width

**Figure 3:**
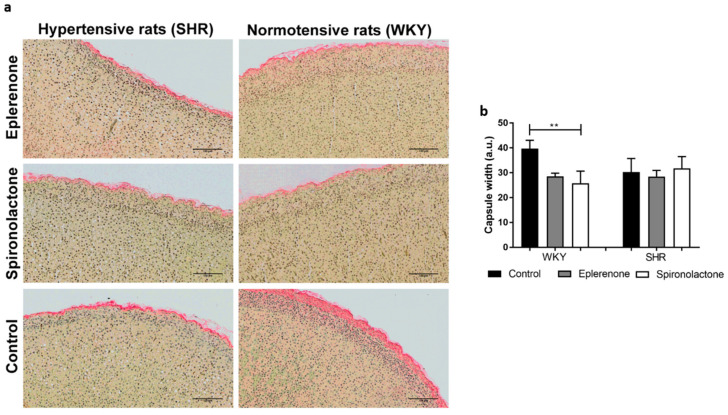
Adrenal glands stained with Sirius red (100×) (**a**) and adrenal capsule width comparison between all the analyzed groups (**b**). ** *p* < 0.01.

There was no significant difference in the width of the adrenal capsule between SHR and WHY rats (Figure 3). Nevertheless, normontensive WKY rats treated with spironolactone had a significantly narrower adrenal capsule (WKY control: 39.72 ± 3.33 vs. WKY spironolactone: 25.80 ± 4.86, *p* < 0.01).

#### 3.3.2. Cell Proliferation

**Figure 4:**
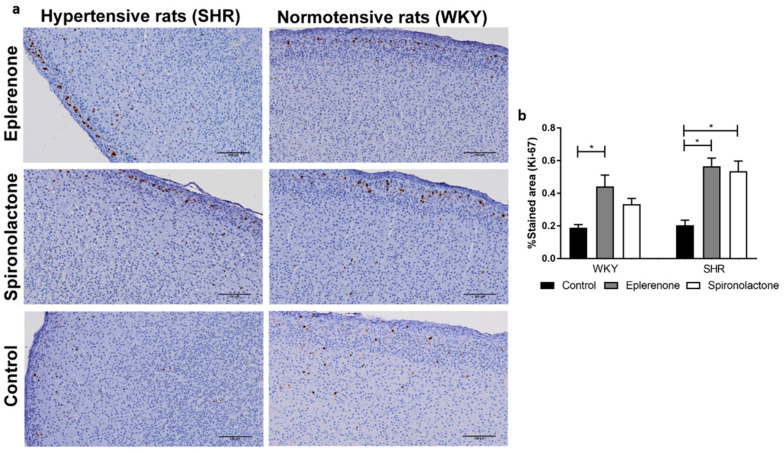
Adrenal glands stained for Ki-67 (100×) (**a**) and percentage of stained area for Ki-67 (**b**). * *p* < 0.05.

Cell proliferation was evaluated by immunohistochemistry staining for Ki-67. In the adrenal cortex, proliferative cells were most abundant in the glomerulosa (Figure 4a).

Proliferation in the cortices of SHR and WKY rats was not significantly different. However, Ki-67 expression was higher in SHR exposed to MRAs, an effect that was both observed after eplerenone (SHR control: 0.20 ± 0.03% vs. SHR eplerenone: 0.57 ± 0.12%; *p* < 0.01) and spironolactone treatment (SHR control: 0.20 ± 0.03% vs. SHR spironolactone: 0.54 ± 0.06%; *p* < 0.05). In WKY normotensive rats, a similar effect was observed only after eplerenone treatment, (WKY control: 0.19 ± 0.02% vs. WKY eplerenone: 0.44 ± 0.07; *p* < 0.05) (Figure 4).

#### 3.3.3. CYP11B1, CYP11B2, and IZA Expression

Adrenal gland CYP11B1, CYP11B2, and IZA expression although higher in most of SHR groups, were not significantly different in either control or treated SHR and WKY rats (*p* > 0.05; Table 1).

#### 3.3.4. Steroid Quantification

**Figure 5:**
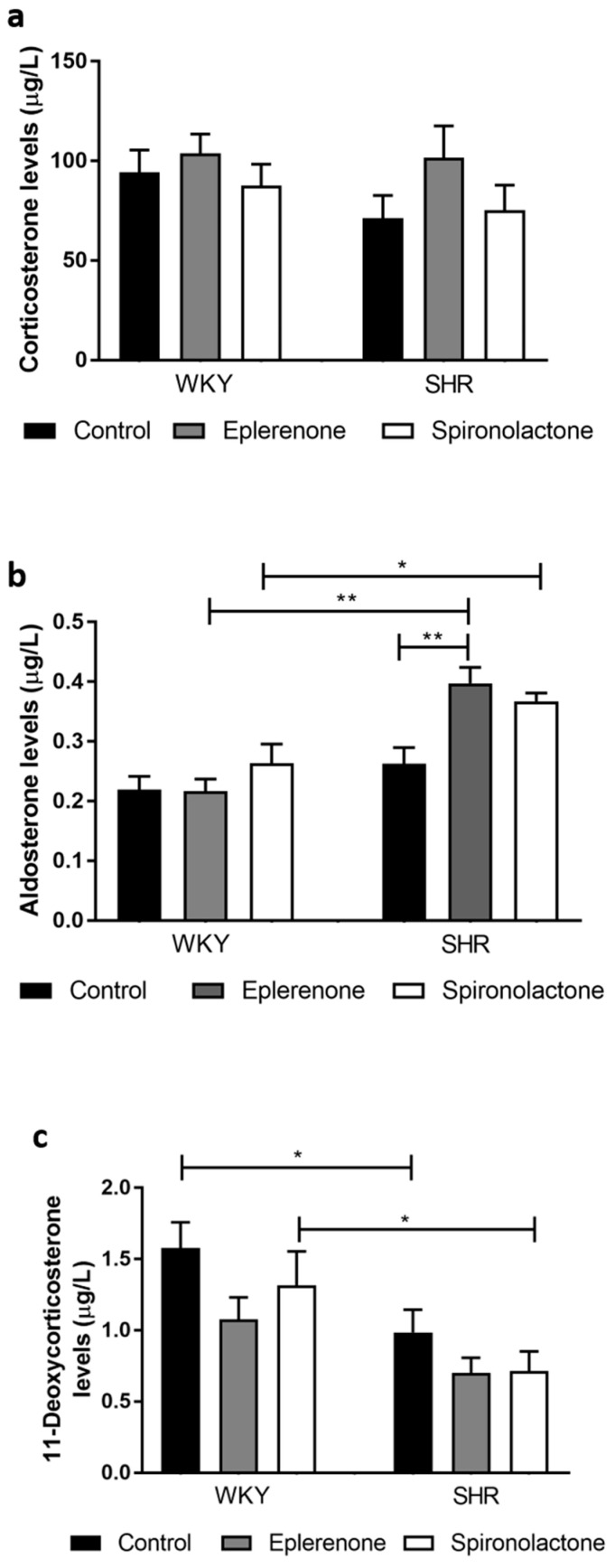
Corticosterone (**a**); aldosterone (**b**); and 11-deoxycorticosterone (**c**) plasma levels in Wistar Kyoto (WKY) and Spontaneous Hypertensive rats (SHR). * *p* < 0.05; ** *p* < 0.01.

There were no significant differences in plasma levels of corticosterone between any of the groups (Figure 5a, *p* > 0.05).

Aldosterone levels were significantly higher in MRA treated SHR as compared to WKY normotensive rats’ (*p* < 0.05; Figure 5b). In addition, aldosterone levels were higher in eplerenone treated SHR as compared to non-treated SHR (SHR control: 0.26 ± 0.03 µg/L vs. SHR eplerenone: 0.40 ± 0.03 µg/L, *p* < 0.01)

Lower 11-deoxycorticosterone levels were observed in untreated SHR compared with non-treated normotensive WKY rats (WKY control: 1.58 ± 0.18 µg/L vs. SHR control: 0.99 ± 0.16 µg/L, *p* < 0.05). Similarly, spironolactone treated SHR rats showed lower 11-deoxycorticosterone levels when compared to spironolactone treated normotensive rats (WKY spironolactone: 1.31 ± 0.24 µg/L vs. SHR spironolactone: 0.72 ± 0.14 µg/L; *p* < 0.05).

## 4. Discussion

MRAs bind to mineralocorticoid receptors to antagonize the action of aldosterone, the endogenous mineralocorticoid. MRAs are a drugs class licensed for the treatment of primary hyperaldosteronism, resistant forms of hypertension, heart failure, nephrotic syndrome, and liver cirrhosis with edema and ascites. Spironolactone and eplerenone are the two MRAs approved by the FDA [2,3,4]. Besides acting on mineralocorticoid receptors, MRAs and most particularly spironolactone, was reported to exert local actions on the adrenal cortex [8,9,10,16,17]. The aim of this research study was to explore the effects of the two MRAs commercially available on the adrenal cortex physiology and morphology of normotensive and hypertensive rats.

One of the most frequently reported effect of spironolactone on the adrenal cortex is a morphological change characterized by the appearance of “spironolactone bodies” within the glomerulosa cells cytoplasm [8,9,10]. The so called “spironolactone bodies” consist of laminated whorls with a central core surrounded by smooth concentric membranes, which are thought to be derived from endoplasmic reticulum [18,19]. Previous studies reported that the “spironolactone bodies” content stains with Luxol fast blue and so this method was appointed as being useful to identify its presence [10,18]. However, using the same technical procedures no morphological changes compatible with the presence of “spironolactone bodies” were identified in the adrenal cortex of normotensive nor hypertensive rats treated either with spironolactone or eplerenone. Since the “spironolactone bodies” were previously described to occur after prolonged spironolactone-treatment in human patients [8,10], the time period that this study lasted not have been enough to induce its appearance. Nevertheless, a period of 28 days of MRAs treatment was enough to achieve blood pressure control in hypertensive rats towards normal values, as well as to induce other morphological and functional alterations in the adrenal cortex of the normotensive and hypertensive rats. However, the morphological alterations observed depicted a different pattern in the normotensive and hypertensive rats suggesting that although SHR rats were originally bred from the WKY rats, there are differences between the two groups in adrenocortical features and response, as well as in blood pressure. Additionally, previous studies reported that SHR and WKY rats exhibit distinct behavioral and neurometabolic features [20,21,22,23].

Arterial blood pressure of normotensive rats was not influenced by the MRAs treatment. Under normal physiological conditions, this phenomenon could be justified by the activation counterregulatory mechanisms that participate in the balance the sodium and water absorption in the kidney, as well as the vascular tonus, thus preventing hypotension [24,25]. Similar mechanisms are observed in hypertensive rats 21 days after MRA treatment initiation, when excess sodium and volume overload are normalized and accompanied by blood pressure levels stabilization at values similar to those observed in normotensive rats. In hypertensive rats there were more lipid droplets in the adrenal cortex cells compared with normotensive rats—these droplets contain cholesterol esters that are the substrates for steroidogenesis [26]. Since hypertensive rats also depict higher aldosterone levels, the accumulation of lipid droplets within the zona glomerulosa may be an adaptive response to the enhanced need of cholesterol esters for aldosterone synthesis. Similar findings regarding the association between lipid droplets density within the glomerulosa and aldosterone levels were reported in other studies [27,28].

Hypertensive rats also showed a higher density of lipid droplets in the zona fasciculata than normotensive rats, but this was not associated with significant differences in circulating corticosterone. Previously, higher basal corticosterone levels were reported in SHR rats than WKY rats, but only in the period preceding the establishment of hypertension [29]. Therefore, higher lipid droplet density within the fasciculata observed in our study could hypothetically represent the persistence of an adaptive phenomenon required to foster enhanced corticosterone production which afterwards does not experience.

Contrary to the aldosterone, findings, 11-deoxycorticosterone levels were mostly lower in SHR groups when compared to WKY. Since 11-deoxycorticosterone is a precursor of both corticosterone and aldosterone, it may be that increased conversion of 11-deoxycorticosterone to aldosterone accounts for this. Although the expression of the steroidogenic enzymes CYP11B1 and CYP11B2 was increased in most of SHR groups when compared with WKY, differences were not statistically significant. This finding is possibly due to the low IHQ accuracy as compared to other molecular approaches, which could be more appropriate to quantify the steroidogenic enzymes in the adrenal cortex.

MRA treatment resulted in noticeable effects on the adrenal cortex morphology. Indeed, signs of higher adrenal cortex cell proliferation were observed upon exposure to both MRAs drugs. As expected, proliferative cells were predominantly located in the glomerulosa layer, since under physiological conditions, proliferating adrenocortical cells are located preferentially in the outermost layer and become apoptotic when the cortical–medullary boundary is reached [30,31].

MRA treatment results in the blockade of aldosterone receptors, which is accompanied by elevated aldosterone levels in hypertensive rats, suggesting the occurrence of a counter regulatory activation of the RAAS with the possible increased angiotensin II secretion known to stimulate aldosterone-secreting cell proliferation [32,33]. Unfortunately, due to limited plasma volume derived from the use of a small rodent in the experiments, it was not possible to measure renin activity and angiotensin II levels to confirm this. Nevertheless, previous data supports this hypothesis since aldosterone receptor blockade with both MRAs drugs, eplerenone and spironolactone, has previously been shown to increase renin activity, angiotensin II and aldosterone levels in hypertensive rats and humans [34,35,36,37]. In addition, our study further suggests that the MRAs effects on the aldosterone production are independent of a direct action on the steroidogenesis pathway, since no differences in the expression of proteins involved in this process were observed. Contrarily, previous in vitro studies, reported that spironolactone interfered with steroidogenesis by inhibiting aldosterone and cortisol production [16,17].

However, it should be noticed that in vitro studies lack the involvement of other key players in aldosterone production, such as the RAAS. From the present literature, it is clear that the RAAS must have a major role, as in the present in vivo experiments and can counteract any direct inhibitory action of the MRAs, leading instead to elevated aldosterone levels [35,37]. In contrast, in vitro studies indicate that eplerenone had no effect on either aldosterone or cortisol production [16].

Normotensive rats treated with spironolactone showed thinner adrenal capsules. Previous studies found that spironolactone reduced the content of the collagen fiber content in other tissues, such as in the myocardium [38,39]. Although, previous studies reported that eplerenone had similar anti-fibrotic effects on the myocardial tissue [40], in our study, eplerenone treatment did not significantly reduce adrenal capsule width. The mechanisms behind the spironolactone effect on the adrenal capsule are still unknown; however, although it is positive in the myocardium context, the consequences of a reduction of the connective tissue of the adrenal gland may compromise the gland structure. The mechanism of this effect and its possible clinical implications requires further investigation.

## 5. Conclusions

In conclusion, exposure to two different MRAs drugs leads to functional and morphological alterations in the adrenal gland. The MRAs effects on the aldosterone levels of hypertensive rats suggest secondary activation of the RAAS, which may be responsible for inducing aldosterone-secreting cell proliferation. This effect seems to be independent of the steroidogenic enzymes’ expression. The clinical and prognostic value of reactive hyperaldosteronism in response to an MRA blockade still requires further investigation.

## Figures and Tables

**Table 1:** Percentage of stained area of aldosterone synthetase (CYP11B2), 11β-hydroxylase (CYP11B1), inner zone antigen (IZA), in the adrenal cortex of Wistar Kyoto (WKY) and Spontaneous Hypertensive rats (SHR).

Groups	CYP11B1 (%)	CYP11B2 (%)	IZA (%)
WKY control	42.42 ± 3.04	2.12 ± 0.70	31.63 ± 2.37
WKY eplerenone	42.14 ± 1.78	4.02 ± 0.34	30.88 ± 2.70
WKY spironolactone	46.01 ± 1.44	3.58 ± 0.71	35.18 ± 2.67
SHR control	49.21 ± 4.15	1.84 ± 0.38	35.08 ± 1.56
SHR eplerenone	43.27 ± 5.26	5.59 ± 2.57	35.19 ± 1.27
SHR spironolactone	51.80 ± 1.23	4.15 ± 1.67	37.77 ± 2.48
*p*-value	NS	NS	NS

## Data Availability

The data presented in this study are available on request from the corresponding author.

## Supplementary Materials

The following are available online at https://www.mdpi.com/article/10.3390/biomedicines9040441/s1, Figure S1: Example of an adrenal cortex of a spironolactone treated hypertensive rats, stained with Luxol fast blue (200×). No spironolactone bodies were identified, Table S1: MRM transitions and MS parameters for each analyzed compound; Table S2: Calibration parameters, recoveries (%), intra-day and inter-day precision (RSD) for steroids quantification analysis.
