# Supplementary material for: Mineralocorticoid Receptor Antagonists Eplerenone and Spironolactone Modify Adrenal Cortex Morphology and Physiology
Source: Biomedicines. 2021 Apr 20;9(4):441. doi: 10.3390/biomedicines9040441 (PMC8074383; doi:10.3390/biomedicines9040441)
Supplement: Supplementary file 1 [file biomedicines-09-00441-s001.zip › biomedicines-1179510-supplementary.pdf]

biomedicines

MDPI

Supplementary Material

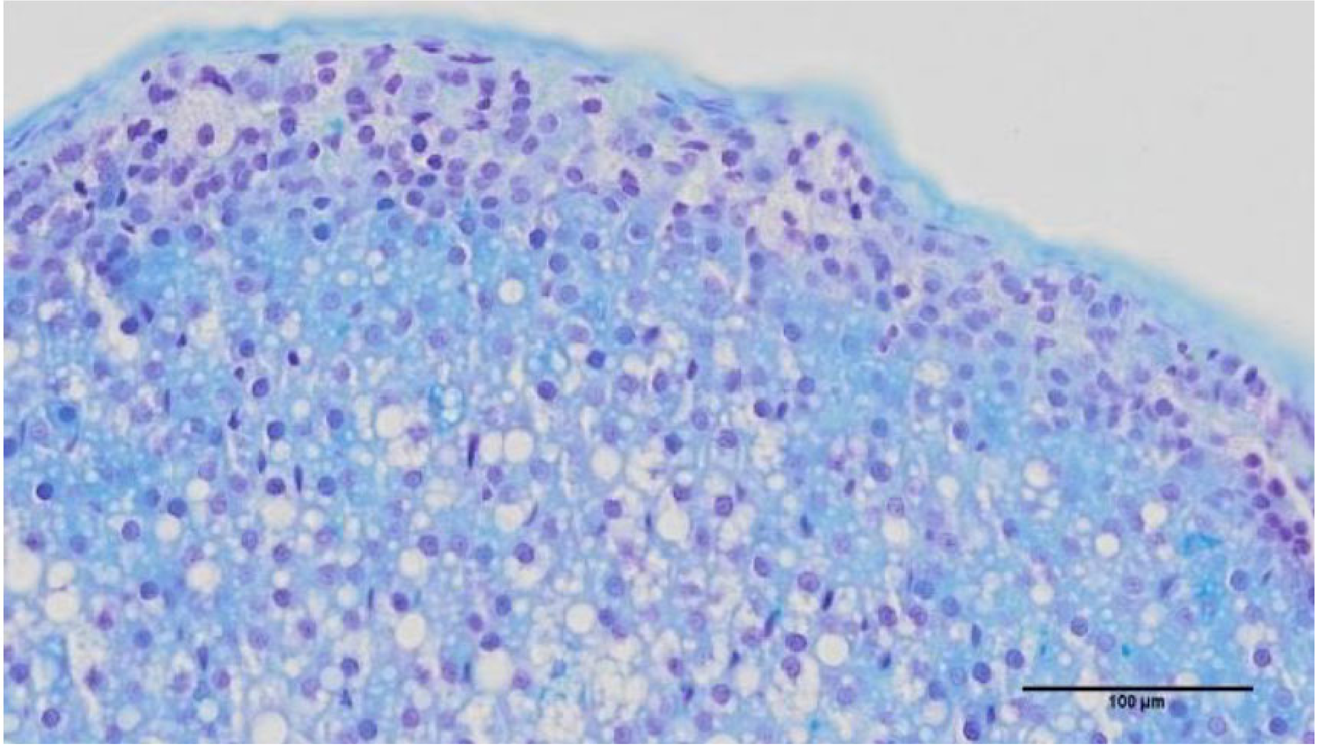

Figure S1 – Example of an adrenal cortex of a spironolactone treated hypertensive rats, stained with Luxol fast blue (200×). No spironolactone bodies were identified.

Table S1. MRM transitions and MS parameters for each analyzed compound.

Compound	Retention Time (min)	MRM Transition (m/z)	Cone Voltage (V)	Collision Energy (V)
Corticosterone	9.70	347.0 > 121.0	30	30
11-deoxycorticosterone	9.95	331.0 > 109.1	30	30
Hydrocortisone (IS)	9.40	363.0 > 327.0	30	20

Table S2. Calibration parameters, recoveries (%), intra-day and inter-day precision (RSD) for steroids quantification analysis.

Compound	b ± Sb	a ± Sa	Sy/x	r2	LOD (µg/L)	LOQ (µg/L)	Recovery %	Intra-day RSD	Inter-day RSD
Corticosterone	52.273 ± 2.964	137.489 ± 0.020	5.440	0.9998	0.31	1.04	104.8	4.3	6.0
11-deoxycorti-costerone	279.216 ± 0.126	−13.517 ± 1.912	23.362	0.9997	0.25	0.64	102.3	4.4	13.5

b - slope, a - intercept, r_2 - correlation coefficient; S(b) and S(a) - standard deviations of slope and intercept, S(y/x) - standard deviation of y-residuals of regression line, LOD—limit of detection; LOQ—limit of quantification; RSD – relative standard deviation.
